# Multiple Antimicrobial Resistance and Heavy Metal Tolerance of Biofilm-Producing Bacteria Isolated from Dairy and Non-Dairy Food Products

**DOI:** 10.3390/foods11182728

**Published:** 2022-09-06

**Authors:** Hasan Ejaz, Kashaf Junaid, Humaira Yasmeen, Amina Naseer, Hafsa Alam, Sonia Younas, Muhammad Usman Qamar, Abualgasim E. Abdalla, Khalid O. A. Abosalif, Naveed Ahmad, Syed Nasir Abbas Bukhari

**Affiliations:** 1 Department of Clinical Laboratory Sciences, College of Applied Medical Sciences, Jouf University, Sakaka 72388, Saudi Arabia; 2 Department of Microbiology and Molecular Genetics, The Women University, Multan 66000, Pakistan; 3 HKU-Pasteur Research Pole, School of Public Health, LKS Faculty of Medicine, The University of Hong Kong, Hong Kong, China; 4 Department of Microbiology, Faculty of Life Sciences, Government College University, Faisalabad 38000, Pakistan; 5 Department of Pharmaceutics, College of Pharmacy, Jouf University, Sakaka 72388, Saudi Arabia; 6 Department of Pharmaceutical Chemistry, College of Pharmacy, Jouf University, Sakaka 72388, Saudi Arabia

**Keywords:** dairy, non-dairy, food contaminants, heavy metals, antimicrobial resistance, biofilm

## Abstract

Foodborne pathogens have acquired the ability to produce biofilms to survive in hostile environments. This study evaluated biofilm formation, antimicrobial resistance (AMR), and heavy metal tolerance of bacteria isolated from dairy and non-dairy food products. We aseptically collected and processed 200 dairy and non-dairy food specimens in peptone broth, incubated them overnight at 37 °C, and sub-cultured them on various culture media. Bacterial growth was identified with biochemical tests and API 20E and 20NE strips. The AMR of the isolates was observed against different antibacterial drug classes. Biofilm formation was detected with the crystal violet tube method. Heavy metal salts were used at concentrations of 250–1500 µg/100 mL to observe heavy metal tolerance. We isolated 180 (50.4%) bacteria from dairy and 177 (49.6%) from non-dairy food samples. The average colony-forming unit (CFU) count for dairy and non-dairy samples was 2.9 ± 0.9 log CFU/mL and 5.1 ± 0.3 log CFU/mL, respectively. *Corynebacterium kutscheri* (n = 74), lactobacilli (n = 73), and *Staphylococcus aureus* (n = 56) were the predominant Gram-positive and *Shigella* (n = 10) the predominant Gram-negative bacteria isolated. The correlation between biofilm formation and AMR was significant (*p* < 0.05) for most cephalosporins, aminoglycosides, and fluoroquinolones. Heavy metal tolerance tended to be higher in biofilm producers at different metal concentrations. The pathogens isolated from dairy and non-dairy food showed a high burden of AMR, high propensity for biofilm formation, and heavy metal tolerance, and pose an imminent threat to public health.

## 1. Introduction

Foodborne diseases are a significant public health concern worldwide [1]. They affect about 600 million people and cause hundreds of thousands of deaths, 40% of which occur among children, every year [2]. However, the extent to which humans are affected by foodborne infections is largely unknown. In most countries, the incidence of foodborne microbial diseases has significantly increased in the last decade. Some foodborne pathogens, for example, *Escherichia coli* O157:H7, *Salmonella typhi*, *Vibrio parahaemolyticus*, *Staphylococcus aureus*, *Clostridium perfringens*, and *Listeria monocytogenes,* are known to generally pollute most commonly consumed foods such as raw milk, dairy products, fruits, vegetables, meat, and seafood [3,4,5]. In 2019, the World Bank reported that lower-middle-income countries annually spend an estimated US $15 billion to treat foodborne illnesses [2].

Milk and dairy products provide excellent growth conditions for many opportunistic pathogenic microorganisms that cause food spoilage and disease [6]. Over the past decades, warnings about microbial food contamination have increased. However, diligent control and protective measures are an enormous task to assure food safety in the farm-to-fork chain [7]. Over the years, there have been noticeable shifts in the major bacterial pathogens compromising dairy food safety. Before the 1900s, bacteria such as *Mycobacterium tuberculosis*, *Bacillus anthracis*, *S. aureus*, and *Streptococcus* spp. were known to be the cause of a significant disease burden through contamination of dairy products [8]. Nonetheless, newly recognized pathogens, such as *L. monocytogenes*, Shiga-toxigenic *E. coli* (STEC), and *Cronobacter*, have emerged in recent years, while *Salmonella* has remained at the forefront as important pathogens in dairy products [4,9,10]. Foodborne pathogens are not only responsible for severe morbidities and mortality but also contribute to antimicrobial resistance (AMR) [11]. The reasons for the emergence of new and resistant foodborne pathogens is multifactorial. Changes in agricultural practices, increased use of antibiotics, microbial adaptation and evolution, increased consumption of raw, undercooked, or processed foods, and climate change are contributing to the selection pressure which leads to the appearance of new and mutated pathogens [12]. The widespread use of antibiotics in food animal production is escalating, resulting in drug-resistant pathogens. Biofilm production by foodborne pathogens is another important factor in drug resistance and leads to many pathogen outbreaks [13]. Microorganisms in a community produce extracellular polymeric substances, which enable them to grow and bind to one another on surfaces, leading to cell adhesion and biofilm formation [14]. The extracellular polymeric substances provide the structural support to these biofilms to persist in the food industry. Biofilm confers a variety of advantages to the microbes in a food processing environment, including mechanical resistance, physical protection, and protection from chemicals, antimicrobials, and disinfectants [15].

A biofilm can persist up to 10 years in a food processing facility despite regular cleaning and sanitation [16]. The control of pathogenic bacteria in the dairy supply chain has remained a significant hurdle to the manufacture of healthy dairy products. Numerous outbreaks of foodborne illnesses have been linked to pathogenic bacteria that can form biofilms. It is estimated that 80% of the microbial infections that occur in the United States are caused by food contamination of biofilm origin [17]. Compared to planktonic bacteria, biofilm-producing bacteria are more resistant to antibiotics [18]. This increased antibiotic resistance is primarily due to reduced drug diffusion through the biofilm matrix and physiological changes in bacteria caused by the biofilm environment. The environmental conditions in most food processing and production settings support the proliferation, growth, and persistence of biofilms on food and contact surfaces [19].

Several studies have found that environmental heavy metal contamination also threatens food production [20,21]. Environmental bacteria can adapt to the presence of heavy metals by developing resistance to heavy metal ions, and the control of bacteria with the combination of antibiotic resistance and heavy metal tolerance is highly difficult [22]. This study elucidated the prevalence of biofilm-producing bacterial isolates in dairy and non-dairy sources. Furthermore, isolates were compared to find a significant link between biofilm production, AMR, and heavy metal tolerance.

## 2. Materials and Methods

### 2.1. Study Design

A total of 200 dairy and non-dairy products were collected from stores and supermarkets in three provincial districts of south Punjab in Pakistan. One hundred pasteurized dairy samples (liquid milk, dry powdered milk, skimmed milk, flavored milk, yogurt, cream, butter, ice cream, lassi, infant powdered milk, mayonnaise, and chicken spread) and 100 non-dairy samples (biscuits, juices, spices, meat, sauces, herbs, vermicelli, noodles, jams, bread, and green tea) were collected.

### 2.2. Specimen Collection and Processing

The samples were collected aseptically. The solid samples were stored in sterile bags and the liquid samples in sterilized screw-capped containers. Each sample was stored at 4 °C and processed within 24 h. Each sample was homogenized in 0.1% peptone water by mixing 25 g of the crushed solid specimen or 25 mL of the liquid specimen with 225 mL of peptone broth [23]. In order to obtain serial decimal dilutions of the homogenized solution up to 10^−6^, the homogenate was further diluted by mixing one part of the homogenate with nine parts of the diluent.

### 2.3. Isolation and Characterization of Bacteria

The nutrient agar plates were spread with 100 µL of suspension from each diluent tube to determine the colony forming units (CFU) in each sample. For enumeration, a colony counter was used to count colonies on the nutrient agar plates. Those plates exhibiting between 30 and 300 colonies were included in the analysis [24,25]. CFU was calculated after incorporating the dilution factor and sample quantity into a raw colony count. The bacterial colonies were identified using selective and enriched culture media plates of the blood, MacConkey (crystal violet), and mannitol salt agar (Liofilchem, Roseto degli Abruzzi, Italy). In order to identify the bacteria, a 100 µL homogenized suspension prepared by mixing the food samples with peptone broth was inoculated on the culture plates as the primary inoculum. We used the four-sector quadrant streaking technique using a platinum loop to obtain pure bacterial colonies cloned from the same precursor. The loop was flamed, cooled, and streaked through the primary inoculum to carry the bacteria to the next sector. The same steps were repeated to streak the organisms from the second to the third and the third to the fourth streak in a zig-zag pattern [26]. The culture plates were incubated overnight at 37 °C and examined for pure growth involving two bacteria and polymicrobial growth based on colony morphology, hemolysis, fermentation of sugars, color, texture, margin, and pigmentation. The majority of the samples produced well-isolated colonies after incubation; however, if a mixed lawn was observed from any sample, the procedure was repeated with a diluted sample. The polyclonal colonies were subcultured on separate culture media to obtain the pure growth of the single isolate. The well-isolated pure colonies were processed for Gram’s staining, biochemical tests (catalase, oxidase, coagulase, gelatin hydrolysis, and sugar fermentation tests) using Bergey’s manual of determinative bacteriology [27], and analytical profile index kits (API 20E and 20NE strips, bioMérieux, France) identified the *Enterobacterales* and non-fermenting GN isolates [28].

### 2.4. Antimicrobial Resistance Testing

We assessed a panel of 18 antibiotics commonly used against GP and GN bacteria. Each isolated bacterial colony was aseptically mixed with normal saline to make a suspension comparable to the 0.5 McFarland standard, which represents approximately 1.5 × 10^8^ CFU/mL of bacteria. The suspended isolates were spread aseptically using a swab on Mueller–Hinton agar (Liofilchem, Roseto degli Abruzzi, Italy), and antibiotic discs (Oxoid, Basingstoke, United Kingdom) were placed on the surface of the culture plate. The antibiotics used were ampicillin (15 µg), cefuroxime (30 µg), cefoxitin (30 µg), ceftriaxone (30 µg), ceftazidime (30 µg), cefotaxime (30 µg), cefepime (30 µg), imipenem (10 µg), meropenem (10 µg), aztreonam (30 µg), piperacillin-tazobactam (100/10 µg), ciprofloxacin (5 µg), levofloxacin (5 µg), colistin (10 µg), co-trimoxazole (1.25/23.75 µg), tigecycline (15 µg), amikacin (30 µg), and gentamicin (10 µg). Culture plates were incubated for 18–24 h at 37 °C. The resistance of bacterial isolates to particular antibiotics was reported based on the inhibition zones [29].

### 2.5. Biofilm Formation Assay

The method of crystal violet staining in polystyrene test tubes was used to check the potential of isolates to form a ring of biofilms. In brief, a loopful of bacteria grown overnight on tryptic soy agar was mixed with 3 mL of tryptic soy broth (TSB) containing 0.25% glucose (Liofilchem, Roseto degli Abruzzi, Italy) and incubated overnight at 180 rpm and 37 °C [30,31]. A 25 µL of this suspension was diluted 100 × by adding 2.5 mL of TSB in polystyrene test tubes followed by incubation for 24–48 h at 37 °C. The tubes were washed three times with phosphate buffer saline (PBS) without disrupting the adherent biofilms. Once the tubes were dried, the attached bacterial cultures were fixed with 99% methanol followed by the addition of a 0.1% crystal violet stain (Sigma-Aldrich, St. Louis, MO, USA) for 8–10 min [31,32]. The excess stain was removed from the tubes by gently washing with 3 mL of PBS. The bacterial biofilms were solubilized with 20% acetone alcohol (Sigma-Aldrich, St. Louis, MO, USA) for 10 min [31], and optical density (OD) was measured at 540 nm. A sterile test tube with TSB was used as a negative control. The OD value was used to determine whether the bacteria were weak (OD ≤ 2), moderate (OD 2–4), or strong (OD ≥4) biofilm formers [32,33].

### 2.6. Heavy Metal Tolerance Testing

Heavy metal tolerance was determined by testing each bacterial isolate on nutrient agar containing various concentrations of each heavy metal. The bacterial isolates were tested against the heavy metal salts of chromium oxide, molybdenum oxide, cadmium chloride, and arsenic chloride (Sigma-Aldrich, St. Louis, MO, USA). Isolates were cultured on agar plates enriched with varying concentrations (250 µg/100 mL to 1500 µg/100 mL) of metal and incubated for 24 h at 37 °C. The presence of growth indicated tolerance of the heavy metals tested. Cultures growing on a previous concentration were transferred to the next higher concentration until the isolate failed to grow [22,34,35].

### 2.7. Statistical Analysis

Statistical analysis was performed using Statistical Package for the Social Sciences (SPSS) v. 24.0 (IBM, Chicago, IL, USA), and figures were generated with GraphPad Prism v. 9.3.1 (GraphPad Software, Inc., San Diego, CA, USA). We performed a descriptive analysis to determine the frequencies and percentages of the qualitative data. The mean CFU was calculated using the t-test, and for this purpose, log transformation was used to ensure normal distribution of the data and homogeneity of variance. The Shapiro-Wilk test was used to confirm the normality of the data. A binomial logistic regression analysis was used to examine the significance of differences in AMR between biofilm producers and non-producers. The significance of differences in AMR between producers and non-producers was investigated using binomial logistic regression analysis.

## 3. Results

### 3.1. Microbial Count in Different Food Types

The food cultures yielded 357 bacterial isolates, of which approximately equal proportions were isolated from dairy and non-dairy food samples. It was evident from the average CFU count that the number of CFU in non-dairy food samples was very high. Our study found a remarkably high number of GP isolates in comparison to GN isolates. More than half of the GP isolates were obtained from dairy sources, whereas all GN isolates prevailed from non-dairy sources (Table 1).

### 3.2. Distribution of Bacterial Isolates in Food Products

The co-occurrence of more than one bacteria was observed in some food specimens. The dairy products showed the presence of different GP bacterial isolates, predominately *Lactobacillus* spp. and *Bacillus subtilis*. The commonly isolated GP bacteria in the non-dairy products were *Corynebacterium kutscheri* and *Lactobacillus* spp. A notable finding was that the percentage of *Staphylococcus aureus* was similar between dairy and non-dairy sources of food. It was found that the three most common GN bacteria isolated from non-dairy food products were *Shigella*, *Proteus mirabilis*, and *Pseudomonas aeruginosa* (Table 2).

### 3.3. Antimicrobial Resistance of Bacterial Isolates

**Figure 1:**
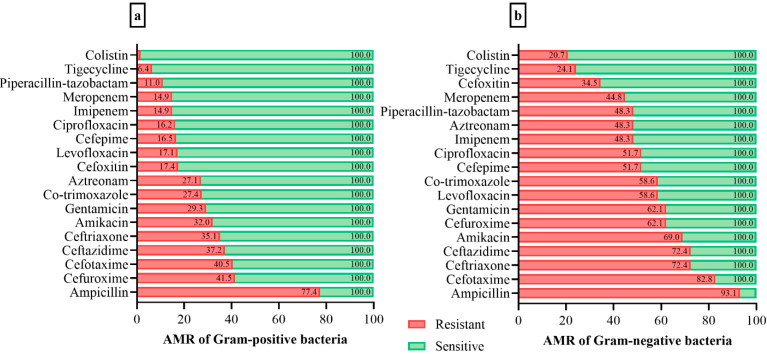
Antimicrobial resistance (AMR) of bacterial isolates from food products. Red bars indicate resistance and green bars susceptibility. (**a**) Gram-positive bacterial isolates; and (**b**) Gram-negative bacterial isolates.

The AMR profile of the GP bacterial isolates demonstrated very high resistance to ampicillin and cefuroxime. The rest of the bacterial isolates also presented resistance to other antibiotics, while a small number showed resistance to colistin and tigecycline. The AMR profile of GN bacterial isolates demonstrated the highest resistance to ampicillin, cefotaxime, ceftriaxone, and ceftazidime. Most of the other bacterial isolates were resistant to several antibiotics, while a few showed low resistance to colistin and tigecycline. The AMR distribution of GP and GN isolates is presented in detail in Figure 1.

### 3.4. Biofilm Production in Bacterial Isolates

The test of isolated bacteria for biofilm formation found that more than two-thirds of the isolates were biofilm producers, with a similar percentage in dairy and non-dairy products. Depending on their ability to form biofilms, these isolates were further divided into weak, medium, and strong biofilm producers. We observed weak, moderate, and strong biofilm production for the isolates in descending order. Substantially more bacterial isolates from non-dairy sources developed biofilms than those from dairy products (Table 3).

**Figure 2:**
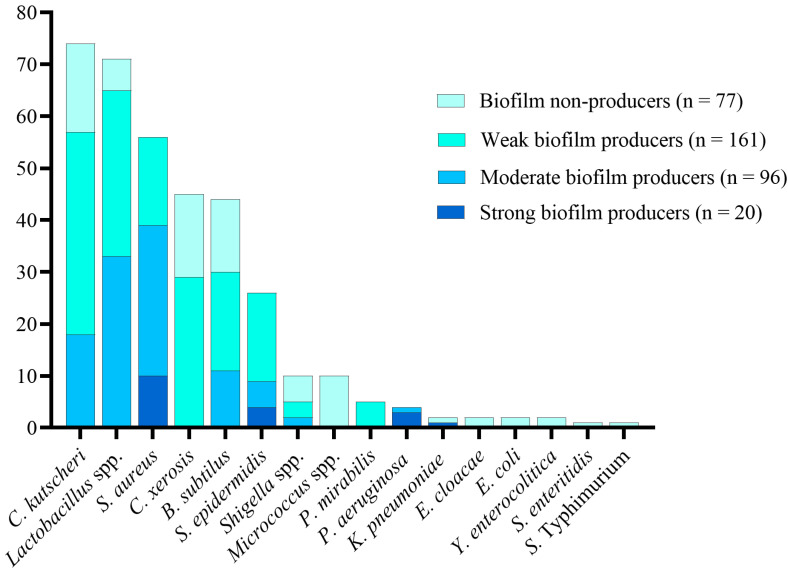
Biofilm production by different bacteria isolated from different food sources. Each bacterial isolate is presented on the x-axis, and the number of biofilm-forming isolates is shown on the y-axis. Different color gradients were used to differentiate between the biofilm non-producers and the different levels of biofilm production.

All *Micrococcus* spp., *Enterobacter cloacae*, *E. coli*, *Yersinia enterocolitica*, *Salmonella* Enteritidis, and *Salmonella* Typhimurium were biofilm non-producers. Strong biofilm formation was predominantly observed in half of *S. aureus* isolates, followed by *Staphylococcus epidermidis*, *P. aeruginosa*, *Lactobacillus* spp., and *Klebsiella pneumoniae*. Moderate biofilm formation was mainly seen in *Lactobacillus* spp. and *S. aureus*. The most frequent weak biofilm producers included *C. kutscheri*, *Lactobacillus* spp., and *C. xerosis* (Figure 2).

### 3.5. Relationship between AMR and Biofilm-Producing and Biofilm-Non-Producing Isolates

Overall, AMR was associated with stronger biofilm producers than non-producers. The correlation between biofilm formation and AMR was statistically significant (*p* < 0.05) for most of the antibiotics, but the correlation was not significant in the case of cefuroxime, cefoxitin, meropenem, colistin, and tigecycline. It is evident from the AMR profile that fewer isolates were resistant to tigecycline and colistin, which presented the lowest level of resistance observed against any antibacterial agents (Table 4).

### 3.6. Assessment of Heavy Metal Tolerance

**Figure 3:**
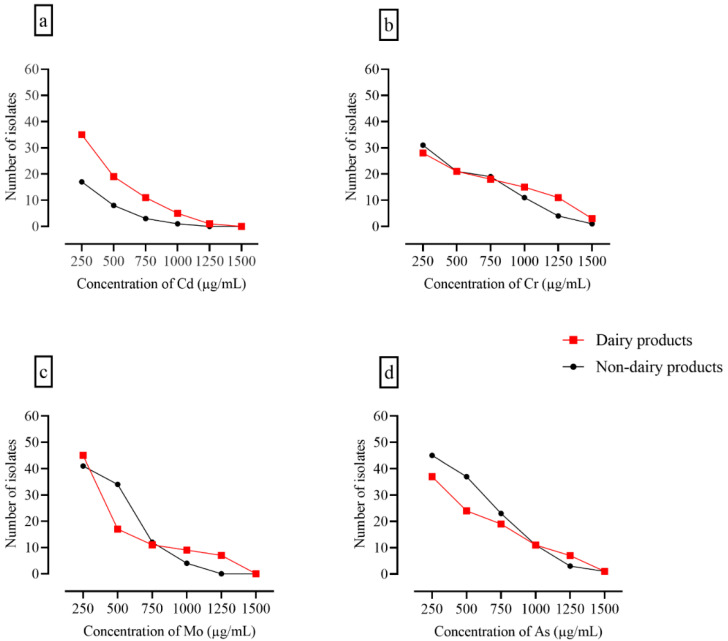
Number of isolates at different concentrations of heavy metals. The panels show the effects of heavy metals on the growth of bacteria isolated from dairy and non-dairy products. Several bacterial cultures did not grow at the lowest concentration of heavy metals (250 µg/100 mL) on nutrient agar. The culture positivity of surviving bacteria grown at the lowest concentration of heavy metals declined with increasing concentrations of; (**a**) Cd: cadmium chloride; (**b**) Cr: chromium oxide; (**c**) Mo: molybdenum oxide; and (**d**) As: arsenic chloride.

**Figure 4:**
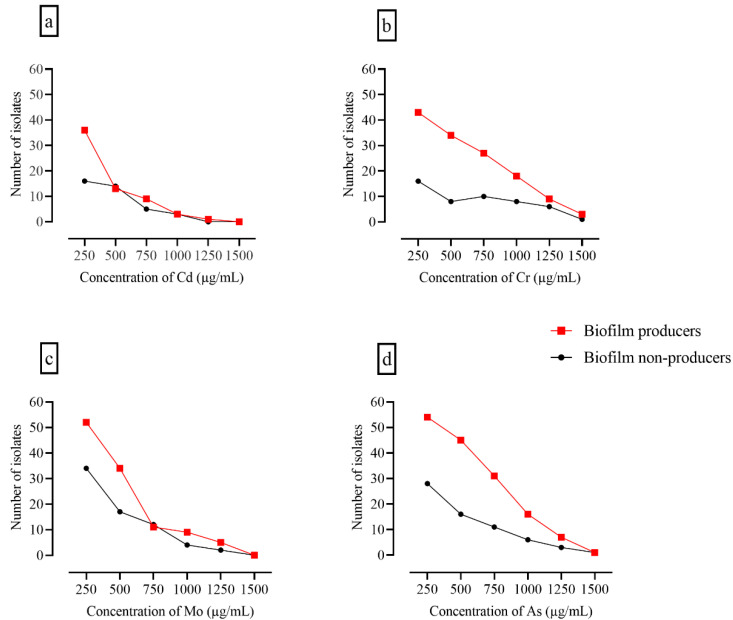
Heavy metal tolerance and biofilm formation. The panels show the heavy metal tolerance of biofilm producers and biofilm non-producers. The number of positive cultures of surviving biofilm producers and biofilm non-producers declined with increasing concentrations of; (**a**) Cd: cadmium chloride; (**b**) Cr: chromium oxide; (**c**) Mo: molybdenum oxide; and (**d**) As: arsenic chloride.

All isolates were analyzed for heavy metal tolerance at metal concentrations ranging from 250 to 1500 µg/100 mL. No significant difference was observed between patterns of heavy metal tolerance. However, the number of bacterial isolates constantly decreased with increasing metal concentration, and a sharp decline was observed at the highest concentration of molybdenum and cadmium. Most isolates that showed heavy metal tolerance also produced biofilms (Figure 3 and Figure 4).

## 4. Discussion

This study describes the presence of multiple AMR in foodborne bacterial isolates with biofilm formation potential, in combination with heavy metal tolerance. Previous studies have indicated that dairy products made from raw milk could be the main source of antibiotic-resistant bacteria that threaten food safety [21,36]. This problem is pervasive in developing countries because of poor food handling methods and hygiene standards, inadequate food safety rules and regulatory systems, and unsatisfactory education of food handlers [37].

Our study reported a high frequency of GP bacterial isolates in dairy products which predominantly include *Lactobacillus* spp., *B. subtilis*, *C. xerosis* and *S. aureus*. None of the GN bacteria were isolated from dairy products. Studies have shown that dairy products contain a high percentage of GP bacteria, though GN isolates have also been detected [38,39]. GP bacteria such as *Lactobacillus* spp. are lactic acid bacteria predominantly present in unpasteurized milk; *C. xerosis* and *S. epidermidis* are frequent contaminants of skin, *S. aureus* is transmitted through animal mastitis, while *B. subtilis* is abundant in the soil, and plant root surfaces more commonly contaminate dairy products [38,40,41]. Raw milk may contain both GP and GN bacteria, and pasteurization destroys most GN bacteria; however, some GP bacteria may survive pasteurization and grow at low temperatures [42], which could be one reason we did not detect GN bacteria. Heat-shock proteins in staphylococcal species allow them to survive when food is heated at 80 °C for 20 min [43,44]. Dairy products may contain GN bacteria more frequently if there is contamination in the water supply. The transfer of GP bacteria to dairy products in Pakistan may be attributed to several factors, such as animal contact outside of forms, poor udder cleaning, and hand milking. Milk is delivered door-to-door by gawalas (milkmen) which facilitates the replication of GP skin contaminants.

The isolation of both GP and GN bacteria from non-dairy food items is consistent with the results of earlier reports [45]. Unhygienic practices may lead to contamination of food products with GP pathogens and heat-resistant bacterial toxins. Diverse bacterial loads have been reported in dairy and non-dairy food products, with the predominant presence of *S. aureus* (25.5%) in milk and dairy products [45]. Another study reported a high prevalence of *S. aureus* (72.2%) in different non-dairy preserved food products [46]. We detected *C. kutscheri* only in non-dairy food products, which is associated with poor hygiene and inadequate hand-washing practices. This bacterium is spread from rat secretions or dung, or waste material of other animals through improper handling of food, e.g., the unwashed hands of infected food handlers, resulting in food contamination [47]. *Shigella* species are well-known foodborne pathogens and are isolated from various food products. In our study, *Shigella* species were isolated only from non-dairy food sources. The presence of *P. mirabilis*, *P. aeruginosa*, *K. pneumoniae*, *E. coli*, and *Salmonella* in food products, especially raw vegetables, has also been reported previously [48,49,50]. These organisms carry plasmid-mediated resistance genes and cause community-acquired human infections. However, in this study, no significant difference in prevalence was observed between these bacterial isolates, consistent with the results of previous studies [21,51].

Microorganisms can grow in the form of biofilm, a physiological state that promotes increased virulence and resistance to conventional antibiotics and sanitizers [52,53]. This study found biofilm formation in two-thirds of the dairy and non-dairy isolates. Our results differed from those of Radovanovic et al. (2020), who reported that 90% of dairy food isolates produced biofilm and that 70% of isolates were moderate biofilm producers [54]. In our study, a few dairy isolates produced strong biofilms, approximately one-fourth moderate biofilms, and nearly one-half weak biofilms. This difference may be attributed to the differences in wavelengths and type of biofilm formation, which can affect the adhesion of bacteria during biofilm formation. A previous study reported that 26.4% of isolates from non-dairy food products were strong biofilm producers, and 25.3% of the isolates had a moderate capacity to produce biofilm, and suggested that biofilms may not play a role in the severity of virulence factors but may contribute to the consistency of infection in the environment [55]. Our analysis of AMR and biofilm-forming potential showed a significant correlation between antibiotic resistance and biofilm formation. Our results showed that AMR was higher in biofilm producers than in biofilm non-producers. Various studies have found a link between biofilm formation and the emergence of antibiotic resistance [56,57].

Heavy metals were often used as chemotherapeutics or antimicrobials in human and animal healthcare during the pre-antibiotic era. Some heavy metals are still being used in agriculture and medical settings. Similar to antibiotic resistance, heavy metal tolerance has rapidly emerged, and microbes with dual antibiotic resistance and heavy metal tolerance are frequently detected. Heavy metal tolerance of food pathogens jeopardizes human health [55,58]. We found potential heavy metal contamination of preserved food samples: however, there was no discernible difference in the pattern of resistance between isolates from different food samples. Nevertheless, heavy metal tolerance was shown by more isolates from non-dairy food products than those from dairy products. The tolerance of chromium, cobalt, arsenic, and molybdenum present in food samples is linked to the increased concentration of these heavy metals in water and soil, which are the main requirements for growing food plants [58]. In Pakistan, only a small number of foods have been tested for heavy metals, and very few comprehensive studies have been conducted to assess their widespread prevalence in foods. A study conducted in the districts of Sialkot and Gujranwala to determine the health risk index (HRI) of heavy metals in different food crops revealed that the highest HRI in wheat samples from the two areas reached values of 8.9 and 9.9, respectively. As a result, the populations of the study areas may face serious health risks, particularly due to wheat consumption [59]. Several other studies have demonstrated varying levels of heavy metals in Pakistani food items [60]. Randhawa et al. reported high levels of cadmium and lead in vegetables, the levels of metals being higher than the maximum residue limit established by the Food and Agriculture Organization (FAO) [61]. Another study seeking to assess direct indicators of food safety and milk quality reported that cadmium, lead, nickel, and copper in dairy milk showed the presence of heavy metals exceeding maximum residue limits (MRLs) as given by the Joint Expert Committee on Food and Agriculture and the World Health Organization [62]. These results in addition to our findings of heavy-metal-tolerant food isolates suggest that the regular monitoring of heavy metals might be useful in preventing unnecessary trace metal buildup in the food chain.

An association between antibiotic resistance and heavy metal tolerance was also observed in this study. Toxic metal ions have become worldwide environmental pollutants due to geochemical cycling and industrial emissions. Microorganisms may use biofilm formation as a strategy to survive the toxic fluxes of these inorganic compounds. The combined action of chemical, physical, and physiological phenomena that are, in some cases, linked to phenotypic variation between the constituent biofilm cells appears to protect biofilm populations from toxic metals [63].

To our knowledge, no previous studies have reported a similar high prevalence of biofilm-producing GP and GN bacterial loads in dairy and non-dairy food products from local markets in Pakistan. Although our study did not investigate AMR genes and heavy metal tolerance genes, our results indicate that the examination of the co-occurrence of and correlations between the two types of genes in biofilm-producing bacterial isolates from food products is an important avenue of future research.

## 5. Conclusions

This study spotlights the contamination of dairy and non-dairy food products by bacterial isolates with multiple antibiotic resistance. The isolation of a high number of biofilm-producing and multiple drug-resistant *S. aureus* is worrisome due to their higher survival rate in the environment. The formation of biofilms and resistance to most cephalosporins, aminoglycosides, and fluoroquinolones were significantly correlated in the isolates investigated in this study, leaving only colistin and tigecycline as effective antimicrobials. The heavy metal tolerance of these bacterial isolates is another dilemma that surpasses the danger of AMR and limits the use of some heavy metals as therapeutic options. To mitigate this problem, the dissemination of highly drug-resistant biofilm-producing bacteria should be prevented, and strict hygiene practices should be employed in all food cycles, including manufacturing, processing, and packaging. The study highlights the importance of field surveillance studies in Pakistan in order to monitor and track emerging and resistant foodborne pathogens.

## Figures and Tables

**Table 1:** Types of food samples and the microbial count of each food source (n = 200).

Type of Food Sample		Number (%) of Bacterial Isolates (n = 357)	CFU/mL (log) Mean ± SD	Number (%) of Gram-Positive Isolates (n = 328)	Number (%) of Gram-Negative Isolates (n = 29)
Dairy food samples (n = 100)	Liquid (n = 44)	180 (50.4%)	2.9 ± 0.9	180 (54.9%)	0 (0%)
	Solid (n = 20)				
	Semisolid (n = 18)				
	Powdered (n = 18)				
Non-dairy food samples (n = 100)	Liquid (n = 20)	177 (49.6%)	5.1 ± 0.3	148 (45.1%)	29 (100%)
	Solid (n = 43)				
	Semisolid (n = 11)				
	Powdered (n = 26)				

**Table 2:** Distribution of Gram-positive and Gram-negative bacterial isolates (n = 357) in dairy and non-dairy food products.

Isolated Bacteria		Number (%) of Isolates from Dairy Food Products (n = 180)	Number (%) of Isolates from Non-Dairy Food Products (n = 177)
Gram-positive isolates (n = 328)	*Corynebacterium kutscheri* (n = 74)	-	74 (41.8%)
	*Staphylococcus aureus* (n = 56)	30 (16.7%)	26 (14.7%)
	*Lactobacillus* spp. (n = 73)	45 (25%)	28 (15.8%)
	*Corynebacterium xerosis* (n = 45)	33 (18.4%)	12 (6.8%)
	*Bacillus subtilis* (n = 44)	44 (24.4%)	-
	*Staphylococcus epidermidis* (n = 26)	18 (10%)	8 (4.5%)
	*Micrococcus* spp. (n = 10)	10 (5.5)	-
Gram-negative isolates (n = 29)	*Shigella* spp. (n = 10)	-	10 (5.6%)
	*Proteus mirabilis* (n = 5)	-	5 (2.8%)
	*Pseudomonas aeruginosa* (n = 4)	-	4 (2.2%)
	*Klebsiella pneumoniae* (n = 2)	-	2 (1.1%)
	*Enterobacter cloacae* (n = 2)	-	2 (1.1%)
	*Escherichia coli* (n = 2)	-	2 (1.1%)
	*Yersinia enterocolitica* (n = 2)	-	2 (1.1%)
	*Salmonella Enteritidis* (n = 1)	-	1 (0.5%)
	*Salmonella Typhimurium* (n = 1)	-	1 (0.5%)

**Table 3:** Types of biofilm produced by bacteria isolated from different food sources (n = 357).

Biofilm Type		Isolates from Dairy Food Products (n = 180)	Isolates from Non-Dairy Food Products (n = 177)
Biofilm non-producers (n = 77)		50 (27.8%)	27 (15.7%)
Biofilm producers (n = 280; 78.4%)	Weak (n = 161; 57.5%)	81 (45%)	80 (44.9%)
	Moderate (n = 99; 35.4%)	43 (23.9%)	56 (31.5%)
	Strong (n = 20; 7.1%)	6 (3.3%)	14 (7.9%)

Total biofilm producers in dairy food products (n = 130/280; 46.4%); total biofilm producers in non-dairy food products (n = 150/280; 53.6%).

**Table 4:** Antimicrobial resistance of biofilm-producing and biofilm-non-producing isolates (n = 357).

Antibiotic	Total Number of Resistant Isolates	Number of (%) Biofilm Producers (n = 280)	Number (%) of Biofilm Non-Producers (n = 77)	*p*-Value
Ampicillin	281	207 (73.7%)	74 (26.3%)	0.002
Cefotaxime	157	135 (86%)	22 (14%)	0.001
Cefuroxime	154	117 (76%)	37 (24%)	0.26
Ceftazidime	143	87 (60.8%)	56 (39.2%)	0.01
Ceftriaxone	136	98 (72.1%)	38 (27.9%)	0.01
Amikacin	125	64 (51.2%)	61 (48.8%)	<0.001
Gentamicin	114	66 (57.9%)	48 (42.1%)	0.001
Co-trimoxazole	107	71 (66.4%)	36 (33.6%)	0.001
Aztreonam	103	61 (59.2%)	42 (40.8%)	<0.001
Levofloxacin	73	71 (97.3%)	2 (2.7%)	<0.001
Cefepime	69	65 (94.2%)	4 (5.8%)	0.01
Ciprofloxacin	68	62 (91.2%)	6 (8.8%)	0.01
Cefoxitin	67	50 (74.6%)	17 (25.4%)	0.27
Imipenem	63	56 (88.9%)	7 (11.1%)	0.05
Meropenem	62	53 (85.5%)	9 (14.5%)	0.23
Piperacillin-tazobactam	50	46 (92%)	4 (8%)	0.02
Tigecycline	28	22 (78.6%)	6 (21.4%)	0.67
Colistin	11	9 (81.8%)	2 (18.2%)	0.65

## Data Availability

Data is contained within the article.
